# Enhanced Surveillance of Sexually Transmitted Infections to Foster a Learning Public Health System

**DOI:** 10.1001/jamanetworkopen.2025.14308

**Published:** 2025-06-17

**Authors:** Harry Reyes Nieva, Jason Zucker, Emma Tucker, Delivette Castor, Michael T. Yin, Peter Gordon, Noémie Elhadad

**Affiliations:** 1 Department of Biomedical Informatics, Columbia University, New York, New York; 2 Department of Medicine, Harvard Medical School, Boston, Massachusetts; 3 Division of Infectious Diseases, NewYork–Presbyterian Hospital/Columbia University Irving Medical Center, New York, New York; 4 College of Physicians and Surgeons, Columbia University, New York, New York

## Abstract

**Importance:**

Sexually transmitted infections (STIs) pose a substantial public health challenge, but fragmented reporting systems have limited capacity for disease surveillance approaches that might support a learning public health system, where near real-time data-driven insights are integrated to inform, evaluate, and adapt public health strategies.

**Objective:**

To examine how health information exchanges and open government data can be leveraged to foster a learning public health system by characterizing and comparing patterns in diagnosis vs testing at a scale commensurate with regional public health agencies.

**Design, Setting, and Participants:**

In this cross-sectional study, chlamydia, gonorrhea, and HIV testing and diagnosis between January 1, 2018, and June 30, 2023, were assessed to study concurrent testing and coinfections and estimate correlates of laboratory testing and positive results. Patient-level electronic health record data from 4 767 322 patients aged 18 years or older spanning multiple health systems in New York City and collected by the public health information exchange Healthix were linked with neighborhood-level information from the American Community Survey.

**Exposures:**

Age, sex, race and ethnicity, socioeconomic status, residential neighborhood, and concurrent STI testing.

**Main Outcomes and Measures:**

Primary outcomes were performance of a laboratory test and test positivity. The viability of using clinical data from public health information exchange to enhance disease surveillance to identify testing gaps, disparities, and important trends, key characteristics of a learning public health system was assessed.

**Results:**

The dataset included 4 767 322 patients (mean [SD] age, 46 [18] years; 61% women) with approximately 33% living in areas of high or very high poverty levels and 66% in areas of low or medium poverty levels. Among 1 519 121 tests for chlamydia, 2% were positive; among 1 574 772 tests for gonorrhea, 1% were positive; and among 1 200 560 tests for HIV, 0.3% were positive. Chlamydia and gonorrhea co-occurred in 1854 cases, representing 7% of chlamydia cases and 21% of gonorrhea cases. Testing behavior was often incongruent with geographic and sociodemographic patterns of incident cases. For example, people living in areas with the highest poverty levels were less likely to be tested for gonorrhea (adjusted odds ratio [AOR], 0.90 [95% CI, 0.89-0.91]; *P* < .001) but almost twice as likely to test positive for gonorrhea (AOR, 1.91 [95% CI, 1.72-2.12]; *P* < .001) compared with those in low poverty areas.

**Conclusions and Relevance:**

This cross-sectional study suggests that health information exchanges and open government data sources may support regional disease surveillance by contributing information not typically available through public health reporting to elucidate inefficiencies and disparities in testing given positivity and coinfection patterns. These and similar insights could be used to continuously drive public health improvements and foster a learning public health system.

## Key Points

**Question:**

Can clinical data collected through public health information exchanges strengthen infectious disease surveillance to facilitate transition toward a learning public health system?

**Findings:**

This cross-sectional study of 4 767 322 participants, representing electronic health records combined from different health systems and open government data, found suboptimal alignment of chlamydia, gonorrhea, and HIV testing and observed incidence by gender, race and ethnicity, age, socioeconomic status, and residential neighborhood; among 1 519 121 tests for chlamydia, 2% were positive; among 1 574 772 tests for gonorrhea, 1% were positive; and among 1 200 560 tests for HIV, 0.3% were positive.

**Meaning:**

This study suggests that health information exchanges may support enhanced surveillance at a scale commensurate with regional public health agencies while enabling analyses not typically possible with traditional data associated with public health reporting.

## Teaser

This cross-sectional study examines whether clinical data collected through public health information exchanges can be leveraged for use in infectious disease surveillance.

## Introduction

Sexually transmitted infections (STIs) pose a substantial public health challenge. In 2023, the Centers for Disease Control and Prevention reported more than 2.4 million incident cases in the US, including 1.6 million cases of chlamydia and more than 600 000 cases of gonorrhea.^1^ Although incidence rates have shown signs of decreasing more recently, for most of the past decade, they were steadily increasing. Less progress has been made among longstanding disparities, however, which largely persist unabated. For example, people who identify as Black or African American comprise 37% of new diagnoses of HIV despite representing only 14% of the population.^2^

Traditionally, prevalence and incidence estimates are derived from surveillance mechanisms that rely heavily on direct notification from health care professionals. As part of standard clinical practice, only positive cases are typically reported to local health agencies, resulting in incomplete information regarding testing and positivity rates. Delays in case reporting, a highly fragmented data infrastructure, and the long lag time required for preparation of surveillance reports further reduce capacity for timely, efficient, and equitable resource allocation.^3,4,5^

A learning public health system refers to an integrated approach that continuously collects and analyzes data from multiple sources to improve public health decision-making and practice.^6^ This system is adaptive, using real-time or near-real-time data to inform policy adjustments, resource allocation, and targeted interventions, ultimately enhancing the responsiveness and effectiveness of public health actions. Surveillance, a foundation of public health practice and policy, may be strengthened using existing, complementary data sources, thus fostering progress toward a learning public health system.^7^ Health information exchanges (HIEs) share electronic health record (EHR) data across health systems within a defined geographic area, allowing for aggregation in a fashion that closely approximates regional surveillance. Health information exchanges also afford comprehensive clinical and demographic information typically unavailable in existing reporting.^8,9^ Use of HIEs has been associated with both cost and time savings on demographic and treatment requests made by public health staff,^10^ a decrease in unnecessary diagnostic testing in emergency departments,^11^ improved continuity of care and patient outcomes,^12,13,14,15^ and reduction of racial and ethnic disparities.^16^ Large public datasets also offer additional context when data linkages are made based on common geography, an important dimension of the social determinants of health.^17,18^

The objective of this study was to demonstrate how near real-time HIE and open government data might be leveraged to characterize and compare patterns in diagnosis vs testing of STIs at a scale commensurate with a regional public health agency, thereby facilitating a transition toward a learning public health system. Specifically, we sought to (1) quantify laboratory tests conducted for chlamydia, gonorrhea, and HIV and incident cases of chlamydia, gonorrhea, and HIV; (2) examine patterns in concurrent testing for chlamydia, gonorrhea, and HIV and co-occurring cases of chlamydia, gonorrhea, and HIV; and (3) characterize population-level differences between testing and positivity along geographic and sociodemographic dimensions.

## Methods

### Study Design, Setting, and Population

We performed a cross-sectional study of adults residing in New York City between January 1, 2018, and June 30, 2023. Laboratory testing included nucleic acid amplification for chlamydia and gonorrhea and serology testing for HIV. We compared patients who received a test for a given STI with patients who were untested, and we compared patients who tested positive for a given STI with patients who tested negative. This study was approved by the institutional review board of Columbia University Irving Medical Center with a waiver of participant consent given the use of deidentified data and the large cohort size. Reporting follows the Strengthening the Reporting of Observational Studies in Epidemiology (STROBE) reporting guideline.

### Data Sources

Our primary data source was Healthix, the largest public HIE in the US. Healthix collects EHR and administrative data from more than 8000 health care facilities for more than 20 million patients using a proprietary system to accurately match data collected for the same patient across multiple care sites.^19^ Healthix provided a limited dataset with participant demographic characteristics, diagnoses, encounters, and laboratory test results. For each participant, we requested information on all chlamydia, gonorrhea, and HIV screening tests available.

We used the Observational Health Data Sciences and Informatics Observational Medical Outcomes Partnership Common Data Model to standardize data from different health systems and facilities.^20^ Cases of HIV were classified using the recommended laboratory HIV testing algorithm.^21^ Laboratory tests for previously known positive cases and HIV viral load monitoring were removed from the dataset. Source values for patient sex may have represented sex assigned at birth or the administrative sex used for billing purposes (which may reflect gender identity). Race and ethnicity source values originated from the electronic health records of health systems that participate in the health information exchange. It is unknown if source values were self-reported or facility designated. Race and ethnicity were also often conflated in the source data, in particular with respect to persons identifying as Hispanic, Latina, Latino, Latine, or Latinx. For brevity, we use *Hispanic or Latino*. We included these variables to provide information about the participants and the generalizability of the results and identify important disparities.

We derived United Hospital Fund neighborhood designations (eTable 1 in Supplement 1) based on grouping of contiguous residential zip code tabulation areas.^22^ The New York City Department of Health and Mental Hygiene commonly uses United Hospital Fund neighborhood designations in surveillance reports. We identified area-based poverty level based on 2020 American Community Survey 5-year estimates.^23^ Area-based poverty level, the percentage of people earning below the federal poverty threshold, is used by the New York City Department of Health and Mental Hygiene to assess socioeconomic status.^24,25^ Using Department of Health and Mental Hygiene standard cut points, we categorized neighborhood-level poverty as low, medium, high, and very high (<10%, 10% to <20%, 20% to <30%, and 30% to 100% of residents, respectively, in zip code tabulation areas living below the federal poverty threshold). Patient ages were grouped into categories based on intervals used in recent New York City Department of Health and Mental Hygiene reports (ie, 20-24, 25-29, 30-34, 35-39, 40-44, 45-49, 50-54, 55-59, 60-64, and ≥65 years).^24^

### Outcomes and Variables

The primary outcomes were performance of a laboratory test and determination of a positive result for chlamydia, gonorrhea, and/or HIV. We examined patterns of concurrent testing, coinfection, and differences based on sociodemographic and geographic factors. We assessed how integrating more comprehensive, timely EHR data from HIEs combined with open government data might improve surveillance capacity, thus operationalizing a core element of a learning public health system. Specifically, we evaluated this integration by examining the ability of enhanced surveillance to elucidate testing gaps and demographic disparities not otherwise or less likely detectable given existing public health reporting practices.

### Statistical Analysis

We computed descriptive statistics and compared proportions using the χ^2^ test and mean values using the *t* test. To assess neighborhood-level clustering of rates, we computed the Moran *I* statistics. To estimate the odds of testing for a given STI or obtaining a positive result, we fit logistic regression models using generalized estimating equations with an exchangeable correlation structure to account for clustering of multiple tests for a given patient. We computed unadjusted and adjusted odds ratios (AORs) and 95% CIs using robust SEs. Models examined sex assigned at birth, race and ethnicity, age, borough of residence, and area-based poverty level. To compute the absolute difference between case and test percentages, we divided the number of tests or cases in each United Hospital Fund neighborhood by citywide totals, then subtracted the percentage of tests performed from the percentage of cases identified (eg, a positive difference indicates more citywide cases than tests in the neighborhood). We conducted all statistical analyses and generated choropleth maps using the R programming environment, version 4.1.1 (R Project for Statistical Computing). All *P* values were from 2-sided tests and results were deemed statistically significant at *P* < .05.

## Results

**Figure 1.**
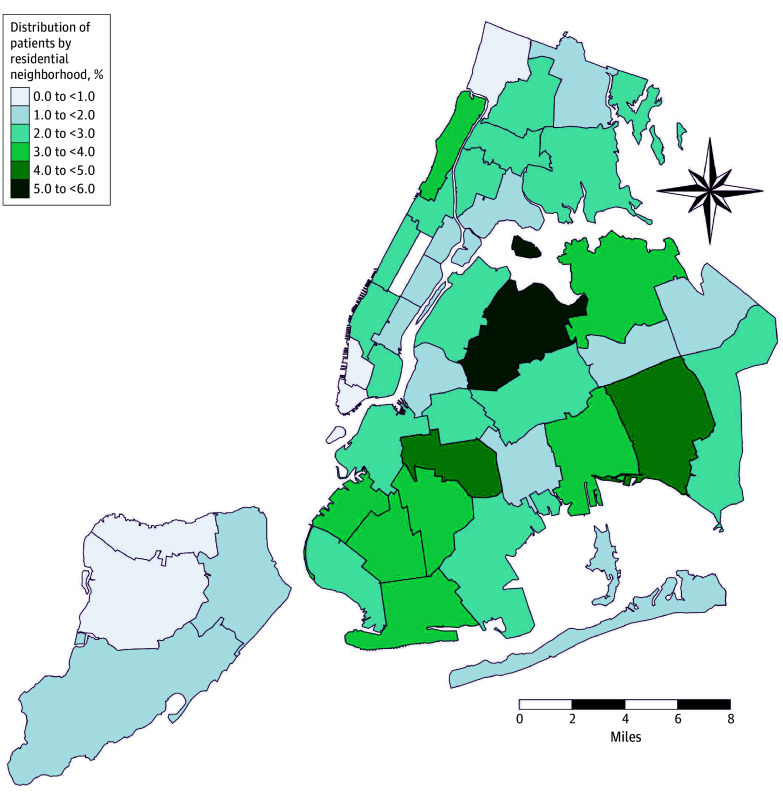
Geographic Distribution of Patients in the Cohort by Residential Neighborhood The source of the shapefile is the New York City Department of Health and Mental Hygeine.^26^ The source of the percentages is the Healthix limited dataset analyzed by the study team and linked to the shapefile based on patient residential zip codes associated with each neighborhood.

The HIE dataset comprised 4 767 322 patients with a mean (SD) age of 46 (18) years; 61% identified as female and 38% as male (Table). A total of 14% of patients identified as Asian or Pacific Islander, 17% as Black or African American, 18% as Hispanic or Latino, 21% as White, and 19% were of unknown race; 11% were labeled as “other” in source data. Generally, 1% to 3% of patients resided in each of the 42 neighborhoods in New York City (Figure 1).^26^ Approximately 66% of patients lived in areas at a low or medium area-based poverty level, and 33% in areas at a high or very high area-based poverty level. Sociodemographic differences in the proportion of persons tested compared with those untested and differences in the proportion of persons with positive test results vs those with negative results were statistically significant.

**Table.** Baseline Characteristics of Patients

Characteristic	Patients (N = 4 767 322), Total No. (%)	Chlamydia, No. (%) of tests		Gonorrhea, No. (%) of tests		HIV, No. (%) of tests	
		Testing (n = 1 519 121)	Positive result (n = 28 251)	Testing (n = 1 574 772)	Positive result (n = 8873)	Testing (n = 1 200 560)	Positive result (n = 3374)
Age, mean (SD), y	46 (18)	36 (13)	27 (9)	36 (13)	29 (10)	39 (15)	42 (14)
Sex^a^							
Female	2 922 650 (61)	1 075 218 (71)	19 299 (67)	1 112 482 (71)	3417 (39)	751 845 (63)	852 (25)
Male	1 806 333 (38)	438 526 (29)	9390 (33)	456 964 (29)	5424 (61)	442 764 (37)	2490 (74)
Unknown	38 339 (1)	5377 (0.4)	59 (0.2)	5326 (0.3)	32 (0.4)	5951 (1)	32 (1)
Race and ethnicity^b^							
Asian or Pacific Islander	674 314 (14)	165 610 (11)	1647 (6)	171 496 (11)	302 (3)	146 649 (12)	86 (3)
Black or African American	807 649 (17)	289 846 (19)	8678 (30)	302 873 (19)	3682 (41)	229 949 (19)	1235 (37)
Hispanic or Latino	850 799 (18)	342 745 (23)	7576 (26)	355 666 (23)	1596 (18)	254 122 (21)	739 (22)
White	1 002 214 (21)	263 821 (17)	2583 (9)	276 510 (18)	1014 (11)	214 989 (18)	392 (12)
Other^c^	525 063 (11)	164 863 (11)	3139 (11)	169 584 (11)	886 (10)	134 870 (11)	352 (10)
Unknown	907 283 (19)	292 236 (19)	5125 (18)	298 643 (19)	1393 (16)	219 981 (18)	570 (17)
Borough of residence							
Bronx	662 357 (14)	271 499 (18)	6421 (22)	272 804 (17)	1921 (22)	194 505 (16)	913 (27)
Brooklyn	1 492 574 (31)	485 206 (32)	8538 (30)	501 624 (32)	2841 (32)	392 844 (33)	1054 (31)
Manhattan	1 457 898 (31)	418 765 (28)	4854 (17)	440 551 (28)	1657 (19)	344 493 (29)	610 (18)
Queens	888 620 (19)	275 909 (18)	7734 (27)	287 022 (18)	2034 (23)	217 985 (18)	692 (21)
Staten Island	265 873 (6)	67 742 (5)	1201 (4)	72 771 (5)	420 (5)	50 733 (4)	105 (3)
Area-based poverty level							
Low (<10% below FPT)	919 691 (19)	247 611 (16)	3972 (14)	264 626 (17)	947 (11)	202 253 (17)	319 (10)
Medium (10% to <20% below FPT)	2 250 976 (47)	676 764 (45)	11 593 (40)	701 128 (45)	3497 (39)	551 405 (46)	1443 (43)
High (20% to <30% below FPT)	959 814 (20)	340 222 (22)	6896 (24)	350 817 (22)	2284 (26)	258 018 (21)	766 (23)
Very high (30% to 100% below FPT)	635 296 (13)	253 967 (17)	6269 (22)	257 622 (16)	2141 (24)	188 455 (16)	843 (25)
Unknown	1545 (0.03)	557 (0.04)	18 (0.1)	579 (0.04)	4 (0.1)	429 (0.04)	3 (0.1)

Abbreviation: FPT, federal poverty threshold.

^a^ The Observational Medical Outcomes Partnership Common Data Model uses the term *gender*, while source data were based on sex; source values may represent sex assigned at birth and/or administrative sex used for billing purposes (which may reflect gender identity, not sex assigned at birth).

^b^ All categories other than “Hispanic or Latino” are exclusively non-Hispanic or non-Latino (eg, non-Hispanic White).

^c^ Derived directly from reporting of “other,” “other race,” or similar in the source data.

### Diagnostic Testing and Positivity Rates

During the review period, patients received 1 519 121 tests for chlamydia, 1 574 772 tests for gonorrhea, and 1 200 560 for HIV (eFigure 1 in Supplement 1). Among tests conducted, 28 251 (2%) were positive for chlamydia (1860 positive per 100 000 tests), 8873 (1%) were positive for gonorrhea (563 positive per 100 000 tests), and 3374 (0.3%) were positive for HIV (281 positive per 100 000 tests).

### Concurrent Testing and Coinfection

Chlamydia testing and gonorrhea testing were largely concurrent (1 489 256 shared tests), comprising 98% of chlamydia tests and 95% of gonorrhea tests (eFigure 2 in Supplement 1). HIV testing in combination with testing for both chlamydia and gonorrhea (n = 530 855) represented 44% of HIV tests, 35% of chlamydia tests, and 34% of gonorrhea tests. Dual testing for HIV and gonorrhea (n = 27 376) was more common than dual testing for HIV and chlamydia (n = 3250). Single testing for HIV comprised 53% of all HIV testing. Testing was positive for both chlamydia and gonorrhea in 1854 tests (7% of chlamydia total cases and 21% of gonorrhea total cases). Results were positive for HIV and chlamydia in 23 tests (1% of HIV total cases and 0.1% of chlamydia total cases), positive for both HIV and gonorrhea in 22 tests (1% of HIV total cases and 0.3% of gonorrhea total cases), and positive for all 3 STIs in 7 tests. One-third of positive chlamydia cases (34%) had an HIV test performed during the same visit (9659 of 28 251 total cases). Nearly one-fifth (22%) of positive gonorrhea cases had an HIV test performed during the same visit (1954 of 8873 total cases). Based on observed coinfection rates, crude projections estimate that approximately 4635 chlamydia cases, 565 gonorrhea cases, and 249 HIV cases were potentially missed among those who were not concurrently tested.

### Testing and Positivity by Population

**Figure 2.**
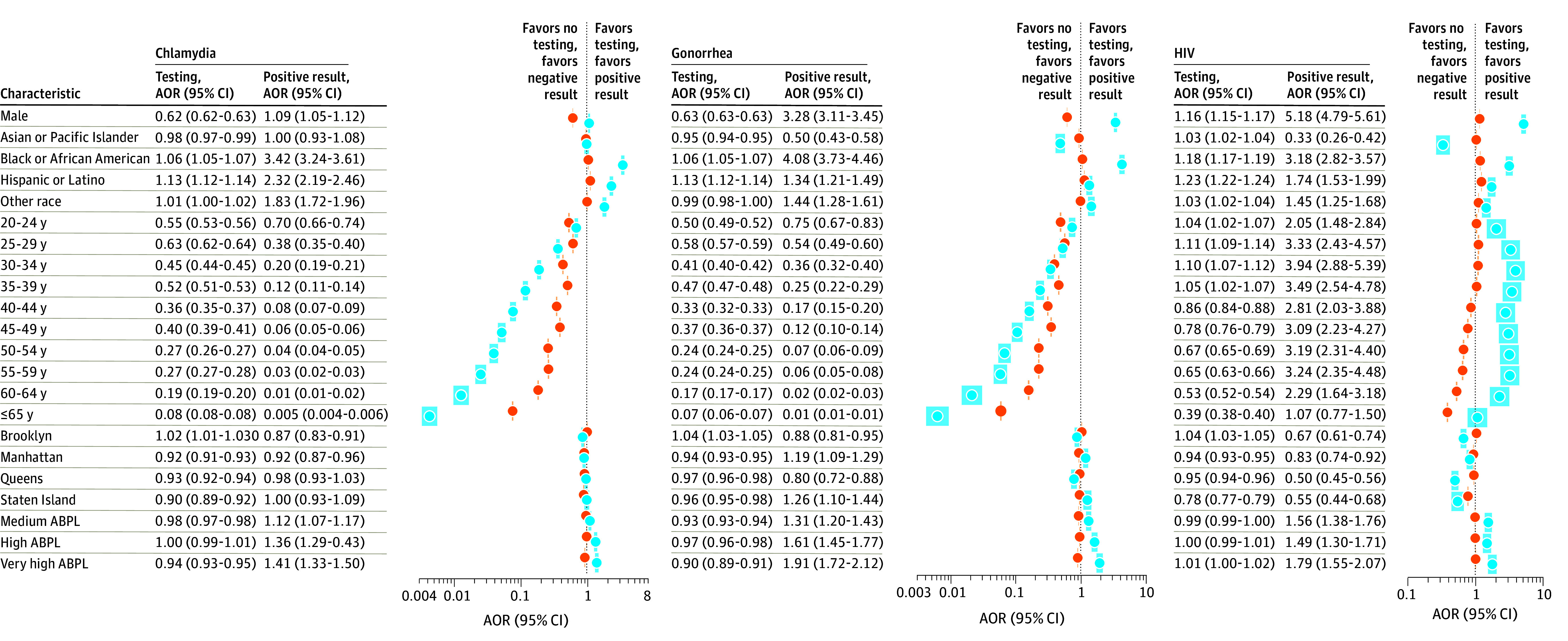
Forest Plots of Adjusted Odds Ratios (AORs) for Laboratory Testing and Positive Test Results by Sexually Transmitted Infection and Patient Characteristics Reference group for sex is female, reference group for race and ethnicity is non-Hispanic White, reference group for age is 18 to 19 years, reference group for borough is Bronx, and reference group for area-based poverty level (ABPL) is low (0% to <10% below federal poverty threshold). The shaded areas around the points indicate 95% CIs.

We identified population-level trends concerning testing behavior and positive cases (Figure 2; eTables 2 and 3 in Supplement 1). Compared with women, men had nearly 40% lower odds of being tested for chlamydia (adjusted odds ratio [AOR], 0.62 [95% CI, 0.62-0.63]) or gonorrhea (AOR, 0.63 [95% CI, 0.63-0.63]) and 16% higher odds of receiving HIV testing (AOR, 1.16 [95% CI, 1.15-1.17]). In contrast, men had 9% increased odds of testing positive for chlamydia (AOR, 1.09 [95% CI, 1.05-1.12]), were 3 times as likely to test positive for gonorrhea (AOR, 3.28 [95% CI, 3.11-3.45]), and 5 times as likely to receive a positive test result for HIV (AOR, 5.18 [95% CI, 4.79-5.61]).

Patients who were Asian or Pacific Islander were nearly as likely to be tested for gonorrhea or HIV as those who were White (gonorrhea: AOR, 0.95 [95% CI, 0.94-0.95]; HIV: AOR, 1.03 [95% CI, 1.02-1.04]) but far less likely to test positive for gonorrhea (AOR, 0.50 [95% CI, 0.43-0.58]) or HIV (AOR, 0.33 [95% CI, 0.26-0.42]) (Figure 2). Participants who were Black or African American had similar odds of being tested for chlamydia, gonorrhea, or HIV compared with those who were White. Nonetheless, their odds of receiving a positive result were 3 to 4 times as high as their odds of taking a test (eg, AOR, 4.08 [95% CI, 3.73-4.46] for a positive gonorrhea test compared with 1.06 [95% CI, 1.05-1.07] for gonorrhea testing). A similar but smaller difference was also seen for chlamydia testing among patients who identified as Hispanic or Latino compared with those who were White (AOR, 1.13 [95% CI, 1.12-1.14] for testing vs 2.32 [95% CI, 2.19-2.46] for positive result).

As age increased, odds of testing and odds of receiving a positive result for chlamydia and gonorrhea decreased. The likelihood of testing for HIV decreased with age as well, although odds of testing positive did not follow this same trend. Compared with patients in the 18- to 19-year-old category, patients who were 30 to 34 years of age had the highest odds of testing positive for HIV (AOR, 3.94 [95% CI, 2.88-5.39]), followed by patients who were 35 to 39 years of age (AOR, 3.49 [95% CI, 2.54-4.78]) (Figure 2).

Differences in odds of testing and positivity were also found among patients given the poverty level of the area in which they lived. Compared with patients residing in areas with low levels of poverty, those residing in areas with high levels of poverty were about as likely to get tested but were more likely to test positive for chlamydia (AOR, 1.36 [95% CI, 1.29-1.43]), gonorrhea (AOR, 1.61 [95% CI, 1.45-1.77]), and HIV (AOR, 1.49 [95% CI, 1.30-1.71]) (Figure 2). In contrast, patients residing in areas with very high levels of poverty were less likely to be tested for either chlamydia (AOR, 0.94 [95% CI, 0.93-0.95]) or gonorrhea (AOR, 0.90 [95% CI, 0.89-0.91]) and more likely to test positive for all 3 STIs (chlamydia: AOR, 1.41 [95% CI, 1.33-1.50]; gonorrhea: AOR, 1.91 [95% CI, 1.72-2.12]; and HIV: AOR, 1.79 [95% CI, 1.55-2.07]).

### Distribution of STIs by Neighborhood

**Figure 3.**
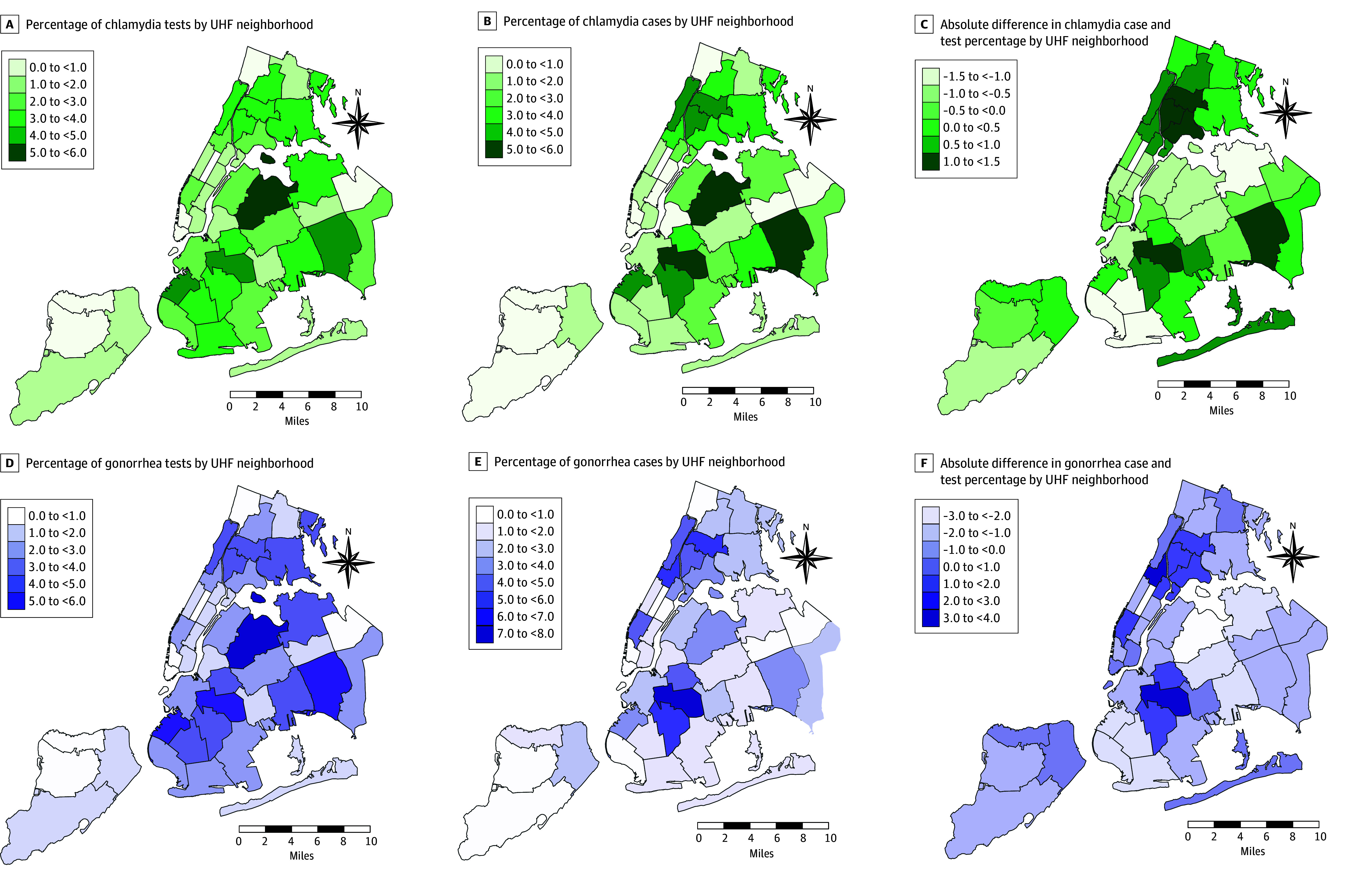
Geographic Distribution of Chlamydia and Gonorrhea Laboratory Testing, Positive Cases, and Absolute Difference in Case and Test Percentage in New York City by Neighborhood, January 2018 to June 2023 The source of the shapefile is the New York City Department of Health and Mental Hygeine.^26^ The source of the percentages is the Healthix limited dataset analyzed by the study team and linked to the shapefile based on patient residential zip codes associated with each neighborhood. UHF indicates United Hospital Fund.

Testing and cases exhibited marked spatial heterogeneity (Figure 3; eFigure 3 and eTable 4 in Supplement 1),^26^ with positive spatial autocorrelation indicating statistically significant levels of clustering in both testing and cases (eTable 5 in Supplement 1). The largest percentage of chlamydia, gonorrhea, and HIV tests were conducted in the West Queens neighborhood (chlamydia tests, 5.9%; gonorrhea tests, 5.8%; and HIV tests, 6.0%). By comparison, the highest percentage of positive cases for chlamydia, gonorrhea, and HIV were in the Bedford Stuyvesant–Crown Heights neighborhood of Brooklyn (chlamydia, 5.9%; gonorrhea, 7.6%; and HIV, 7.8%), compared with West Queens (chlamydia, 5.1%; gonorrhea, 3.5%; and HIV, 3.9%).

The largest positive absolute differences between case and test percentages for chlamydia, gonorrhea, and HIV were consistently found in the Bedford Stuyvesant–Crown Heights neighborhood of Brooklyn (eTable 5 in Supplement 1). Chlamydia comprised 1.4% more citywide positive cases than the percentage of citywide tests, 3.0% more gonorrhea citywide cases than citywide tests, and 3.1% more HIV citywide cases than citywide tests. The largest negative absolute difference between case and test percentages for chlamydia was in the Borough Park neighborhood of Brooklyn (−1.4%). For gonorrhea, the largest negative absolute difference was in West Queens (−2.3%). The Flushing neighborhood of Queens had the largest negative absolute difference for HIV (−2.4%).

## Discussion

We established a robust, scalable data infrastructure to enhance STI surveillance, enable examination of population-level trends, and promote more effective and equitable testing and intervention by leveraging a regional HIE with detailed, near real-time EHR data standardized across health systems. Our findings provide valuable information on laboratory-confirmed cases of chlamydia, gonorrhea, and HIV; underlying testing patterns; and differences across demographic population markers and geographies. The capacity to discern these patterns of misalignment and disparity aligns directly with learning public health system objectives to continuously generate new insights from data and adapt strategies to address evolving public health needs.

The high burden of STIs, demonstrated by the large number of cases, underscores the importance of effective, timely surveillance and targeted interventions. Traditional surveillance relies heavily on direct notification from clinicians and laboratories, which limits understanding of population-level risk and the efficacy and equity of public services and programs. By incorporating data from HIEs and public datasets with neighborhood-level information relevant to social determinants of health, we were able to overcome these limitations and gain more comprehensive insights into STI testing and positivity rates.

Most patients had concurrent testing for chlamydia and gonorrhea, as the most common nucleic acid amplification test performed is capable of identifying both diseases simultaneously. We did not, however, note similarly substantial concurrent testing for HIV in tandem with tests for chlamydia and gonorrhea. This finding underscores the need for implementing and strengthening integrated strategies that simultaneously screen for multiple STIs, as coinfections can have serious health consequences and contribute to ongoing transmission.^27,28,29^ Under the Patient Protection and Affordable Care Act, testing for certain STIs, including HIV, chlamydia, and gonorrhea, must be covered as preventive health benefits by most insurers.^30^ By identifying the occurrence of concurrent testing and co-occurring infections, public health agencies can enhance their strategies for STI prevention and control by addressing missed opportunities for testing and implementing interventions to reduce repeated visits to seek care and the burden of multiple infections.

Enhanced surveillance also revealed disparities in the distribution and alignment between STI testing and positive cases by sociodemographic groups. Consistent with general trends from previous studies, we found that members of certain populations were disproportionately affected by STIs. Our study findings can inform prioritization of strategies and interventions to reduce disparities in the burden of infection, as well as infections overall, in a timely, comprehensive, and precise way.

Differences were also found when comparing testing and positivity rates based on patient sex, race and ethnicity, age, neighborhood, and socioeconomic status. Men were less likely to undergo testing for chlamydia and gonorrhea but had higher odds of receiving a positive result. This finding suggests potential missed opportunities for testing among men. It does not, however, lead us to recommend that women receive less testing, as STIs are more commonly asymptomatic in women. Similarly, patients who identified as Asian or Pacific Islander were almost as likely to be tested for HIV but far less likely to test positive compared with people who identified as non-Hispanic White. People who identified as Black or African American and as Hispanic or Latino were more likely to receive certain tests and test positive. Nonetheless, relative differences between testing and case rates signal possible undertesting. Although STI testing generally decreased as age increased, odds of testing positive for HIV did not follow this trend. Differences suggest missed opportunities for testing. These findings highlight the complex interplay of sociodemographic factors in STI testing and positivity rates, emphasizing the importance of tailored interventions and culturally competent care. By using HIE data, public health agencies may better identify and address disparities and tailor interventions to the specific needs of different populations.

Geospatial analysis demonstrated marked heterogeneity in testing behavior and cases across neighborhoods and clustering of testing and cases. This finding suggests that geography was a key factor among patterns of infection, diagnosis, and treatment, which highlights the importance of the well-established practice of considering geographic factors in addition to demographic factors for STI prevention and control strategies. We recognize that New York City district public health offices were established in 2003 at the borough level for regional allocation of resources. Our primary aim in presenting these expected results is to demonstrate the utility of HIEs as a data source in characterizing the spatial distribution of STIs to help identify high-risk areas, distinguish between subpopulations, and allocate resources more effectively and equitably (eg, targeting interventions to specific areas via mobile testing vans, temporary campaigns in community centers, or local media ads to promote testing and access to services).

It is widely reported that fragmented data collection systems at the state and local levels have been underfunded for years, and it has been estimated that modernizing nonfederal public health reporting systems would require $8 billion per year for 10 years.^4^ In the era of big data, HIEs present opportunities to further empower public health professionals to conduct their work with real-time access to rich EHR data.^7^ This is in contrast to traditional surveillance reporting, which is often delayed and requires that clinicians actively choose to transmit case reports to local public health authorities. The timeliness of HIE data may be especially informative during public health emergencies that require swift attention (eg, the mpox outbreak in New York City during 2022).^31^ Decision processes that are informed by an HIE data system can accommodate varying levels of temporal frequency as needed. By continuously learning from these existing, complementary sources of more timely and comprehensive clinical evidence, public health agencies may adapt strategies and responses to evolving STI dynamics more quickly, ultimately improving outcomes and reducing the burden on communities. This data-driven approach aligns closely with a learning health system,^31^ which can extend to public health agencies operating at a population level instead of an individual service level.^32^ A learning public health system might encompass multiple learning health systems and traditional health systems and can routinely analyze large-scale datasets, extract insights regarding population health, and inform public health practice to enable continual improvement. Implementing a learning public health system framework using integrated health system data could substantially enhance public health preparedness and responsiveness, especially during emerging infectious disease threats and outbreaks. Investment in learning public health system infrastructure could not only improve STI management but more broadly strengthen public health resilience and equity.

### Limitations

We acknowledge several limitations to our study. First, Healthix data did not capture all testing within New York City. Without a comprehensive list of non-Healthix facilities, we were unable to determine which sites were missing. Populations less well represented may have received testing at nonparticipating facilities. In part, this may explain why our dataset had a higher percentage of women (61%) compared with the city population (53%), although it is well known that women have higher levels of health care utilization.^33^ We also used residential zip codes to perform unadjusted geospatial analyses. Although our limited dataset allowed for spatial resolution only to the zip code level, full residential addresses are available to the HIE. Thus, public health agencies may be able to access such information if needed to ensure more precise, targeted intervention. A total of 20% of patients were missing data on race and ethnicity. An additional 10% were only assigned to “other” race and ethnicity in the source data. Although these concerns are commonly found among EHR data, they remain a challenge if we are to support the elucidation of disparities and offer actionable findings. In addition, patients receiving preexposure prophylaxis for HIV prevention often undergo regular STI testing. Although our modeling accounts for repeated measures from the same patient, our approach does not fully address how this may influence findings. Last, our analysis evaluated patterns related to chlamydia, gonorrhea, and HIV. Although our approach to leveraging HIE data would remain largely unchanged (eg, data standardization and linkage), future work is still necessary to tailor analyses to specific use cases and establish that other STIs reflect similar trends. For example, our analysis did not include syphilis because determining infection status and disease stage depends on interpreting multiple, sometimes sequential, laboratory tests in conjunction with clinical information and historical testing, making accurate classification difficult with laboratory test data alone.

## Conclusions

This cross-sectional study of STI testing and diagnosis in New York City illustrates how existing clinical data might support an underresourced public health data infrastructure through HIEs, data harmonization, integration of open datasets, and geospatial analysis. Improving surveillance capacity may offer a more nuanced understanding of population- and neighborhood-level patterns, elucidate inequity, inform targeted intervention, and improve resource allocation. Further collaboration between public health agencies, clinicians, and HIEs are needed to develop fully robust, data-driven approaches and realize this vision of a learning public health system.

## Notes

Supplement 1. **eFigure 1.** STROBE (Strengthening the Reporting of Observational Studies in Epidemiology) Statement Flowchart **eFigure 2.** Concurrent Laboratory Testing and Co-occurring Cases of Chlamydia, Gonorrhea, and HIV in New York City, January 2018-June 2023 **eFigure 3.** Geographic Distribution of HIV Laboratory Testing, Positive Cases, and Absolute Difference in Case and Test Percentage in New York City by Neighborhood, January 2018-June 2023 **eTable 1.** United Hospital Fund (UHF) Neighborhood Zip Code Tabulation Area (ZCTA) Index **eTable 2.** Adjusted Odds Ratios for Laboratory Testing and Positive Test Results by Sexually Transmitted Infection and Patient Characteristics **eTable 3.** Unadjusted Odds Ratios for Laboratory Testing and Positive Test Results by Sexually Transmitted Infection and Patient Characteristics **eTable 4.** Neighborhood-Level Spatial Autocorrelation of Laboratory Testing and Positive Test Results **eTable 5.** Citywide Percentages of Laboratory Tests and Positive Results by Neighborhood

Supplement 2.
Data Sharing Statement
