# Supplementary material for: Enhanced Surveillance of Sexually Transmitted Infections to Foster a Learning Public Health System
Source: JAMA Netw Open. 2025 Jun 17;8(6):e2514308. doi: 10.1001/jamanetworkopen.2025.14308 (PMC12175029; doi:10.1001/jamanetworkopen.2025.14308)
Supplement: Supplement 1. — eFigure 1. STROBE (Strengthening the Reporting of Observational Studies in Epidemiology) Statement Flowchart eFigure 2. Concurrent Laboratory Testing and Co-occurring Cases of Chlamydia, Gonorrhea, and HIV in New York City, January 2018-June 2023 eFigure 3. Geographic Distribution of HIV Laboratory Testing, Positive Cases, and Absolute Difference in Case and Test Percentage in New York City by Neighborhood, January 2018-June 2023 eTable 1. United Hospital Fund (UHF) Neighborhood Zip Code Tabulation Area (ZCTA) Index eTable 2. Adjusted Odds Ratios for Laboratory Testing and Positive Test Results by Sexually Transmitted Infection and Patient Characteristics eTable 3. Unadjusted Odds Ratios for Laboratory Testing and Positive Test Results by Sexually Transmitted Infection and Patient Characteristics eTable 4. Neighborhood-Level Spatial Autocorrelation of Laboratory Testing and Positive Test Results eTable 5. Citywide Percentages of Laboratory Tests and Positive Results by Neighborhood [file jamanetwopen-e2514308-s001.pdf]

Supplementary Online Content

Reyes Nieva H, Zucker J, Tucker E, et al. Surveillance of HIV and other sexually transmitted infections in a learning public health system. *JAMA Netw Open.* 2025;8(6):e2514308. doi:10.1001/jamanetworkopen.2025.14308

eFigure 1. STROBE (Strengthening the Reporting of Observational Studies in Epidemiology) Statement Flow Chart
eFigure 2. Concurrent Laboratory Testing and Co-occurring Cases of Chlamydia, Gonorrhea, and HIV in New York City, January 2018-June 2023
eFigure 3. Geographic Distribution of HIV Laboratory Testing, Positive Cases, and Absolute Difference in Case and Test Percentage in New York City by Neighborhood, January 2018-June 2023
eTable 1. United Hospital Fund (UHF) Neighborhood Zip Code Tabulation Area (ZCTA) Index
eTable 2. Adjusted Odds Ratios for Laboratory Testing and Positive Test Results by Sexually Transmitted Infection and Patient Characteristics
eTable 3. Unadjusted Odds Ratios for Laboratory Testing and Positive Test Results by Sexually Transmitted Infection and Patient Characteristics
eTable 4. Neighborhood-Level Spatial Autocorrelation of Laboratory Testing and Positive Test Results
eTable 5. Citywide Percentages of Laboratory Tests and Positive Results by Neighborhood

This supplementary material has been provided by the authors to give readers additional information about their work.

© 2025 Reyes Nieva H et al. *JAMA Network Open.*

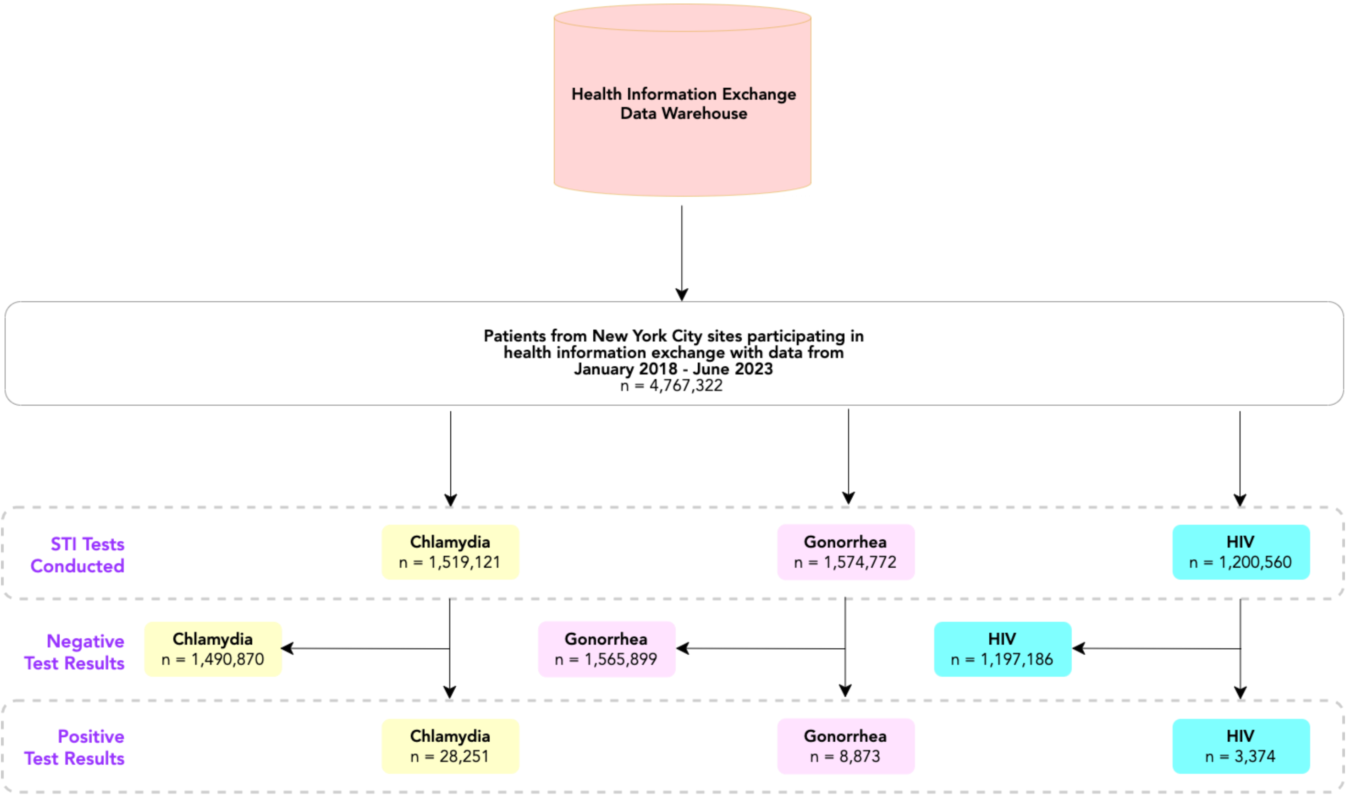

eFigure 1. STROBE (Strengthening the Reporting of Observational Studies in Epidemiology) Statement Flow Chart

© 2025 Reyes Nieva et al. *JAMA Network Open.*

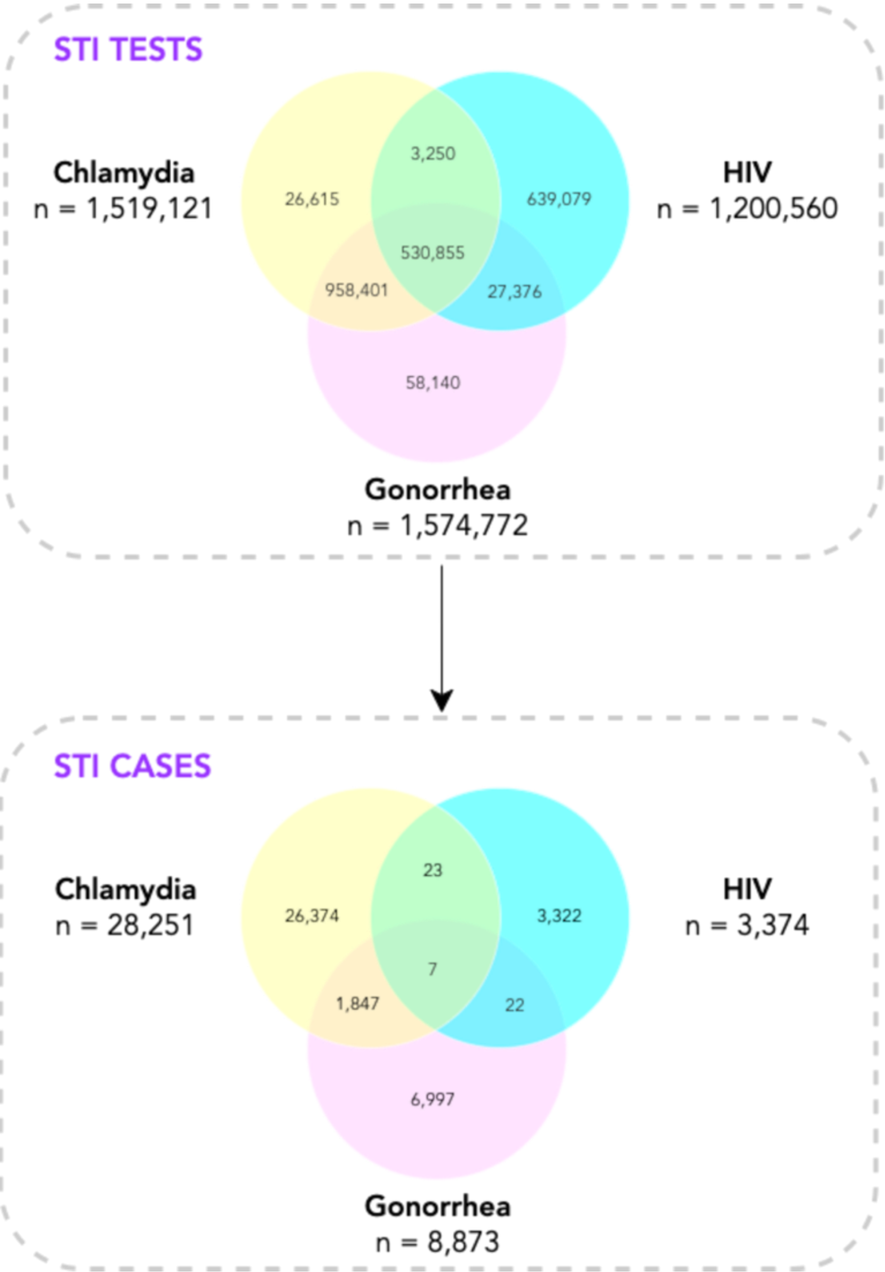

eFigure 2. Concurrent Laboratory Testing and Co-occurring Cases of Chlamydia, Gonorrhea, and HIV in New York City, January 2018-June 2023

© 2025 Reyes Nieva et al. *JAMA Network Open.*

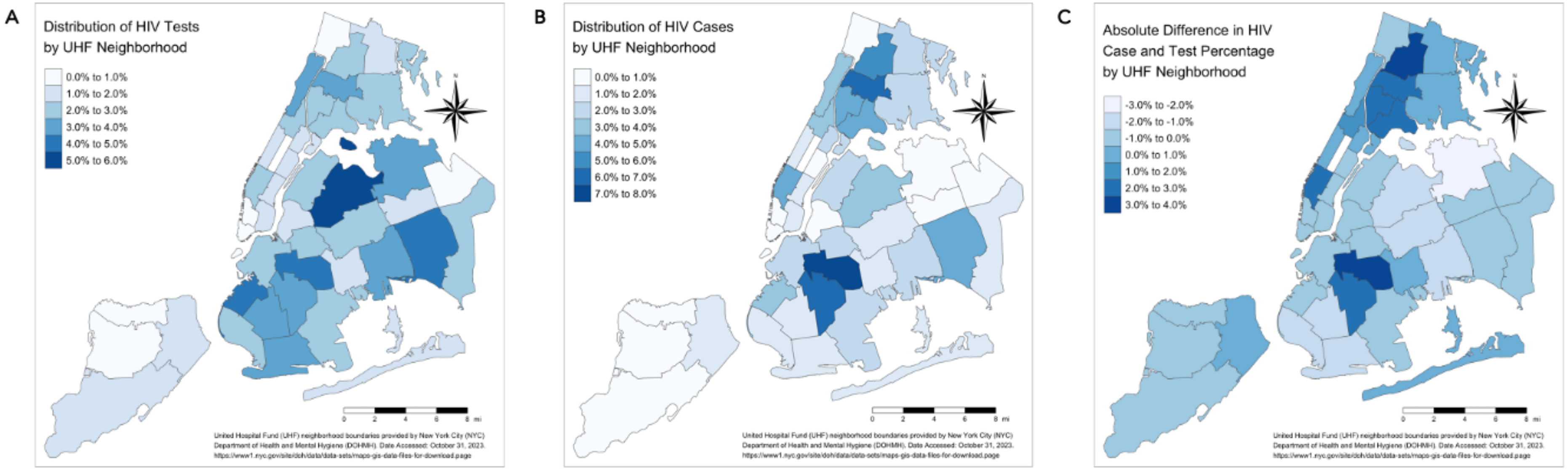

eFigure 3. Geographic Distribution of HIV Laboratory Testing, Positive Cases, and Absolute Difference in Case and Test Percentage in New York City by Neighborhood, January 2018-June 2023

© 2025 Reyes Nieva et al. *JAMA Network Open.*

eTable 1. United Hospital Fund (UHF) Neighborhood Zip Code Tabulation Area (ZCTA) Index

Borough	UHF Code and Neighborhood Name	ZIP Code Tabulation Areas (ZCTAs)
Bronx	101 Kingsbridge - Riverdale	10463, 10471
Bronx	102 Northeast Bronx	10464, 10466, 10469, 10470, 10475
Bronx	103 Fordham - Bronx Park	10458, 10467, 10468
Bronx	104 Pelham - Throgs Neck	10461, 10462, 10465, 10472, 10473
Bronx	105 Crotona - Tremont	10453, 10457, 10460
Bronx	106 High Bridge - Morrisania	10451, 10452, 10456
Bronx	107 Hunts Point - Mott Haven	10454, 10455, 10459, 10474
Brooklyn	201 Greenpoint	11211, 11222
Brooklyn	202 Downtown - Heights - Park Slope	11201, 11205, 11215, 11217, 11231
Brooklyn	203 Bedford Stuyvesant - Crown Heights	11212, 11213, 11216, 11233, 11238
Brooklyn	204 East New York	11207
Brooklyn	205 Sunset Park	11208, 11220, 11232
Brooklyn	206 Borough Park	11204, 11218, 11219, 11230
Brooklyn	207 East Flatbush - Flatbush	11203, 11210, 11225, 11226
Brooklyn	208 Canarsie - Flatlands	11234, 11236, 11239
Brooklyn	209 Bensonhurst - Bay Ridge	11209, 11214, 11228
Brooklyn	210 Coney Island - Sheepshead Bay	11223, 11224, 11229, 11235
Brooklyn	211 Williamsburg - Bushwick	11206, 11221, 11237
Manhattan	301 Washington Heights - Inwood	10031, 10032, 10033, 10034, 10040
Manhattan	302 Central Harlem - Morningside Heights/ Manhattan	10026, 10027, 10030, 10037, 10039, 10115
Manhattan	303 East Harlem	10029, 10035
Manhattan	304 Upper West Side	10023, 10024, 10025, 10069
Manhattan	305 Upper East Side	10021, 10028, 10044, 10128, 10075, 10065, 10162
Manhattan	306 Chelsea - Clinton	10001, 10011, 10018, 10019, 10020, 10036, 10199, 10119, 10111
Manhattan	307 Gramercy Park - Murray Hill	10010, 10016, 10017, 10022, 10110, 10170, 10154, 10153, 10174, 10165, 10168, 10167

© 2025 Reyes Nieva et al. *JAMA Network Open.*

Manhattan	308 Greenwich Village - SoHo	10012, 10013, 10014
Manhattan	309 Union Square - Lower East Side	10002, 10003, 10009, 10278
Manhattan	310 Lower Manhattan	10004, 10005, 10006, 10007, 10038, 10280, 10282, 10271
Queens	401 Long Island City - Astoria	11101, 11102, 11103, 11104, 11105, 11106, 11109
Queens	402 West Queens	11368, 11369, 11370, 11372, 11373, 11377, 11378, 11371
Queens	403 Flushing - Clearview	11354, 11355, 11356, 11357, 11358, 11359, 11360, 11351
Queens	404 Bayside - Little Neck	11361, 11362, 11363, 11364
Queens	405 Ridgewood - Forest Hills	11374, 11375, 11379, 11385
Queens	406 Fresh Meadows	11365, 11366, 11367
Queens	407 Southwest Queens	11414, 11415, 11416, 11417, 11418, 11419, 11420, 11421, 11424
Queens	408 Jamaica	11412, 11423, 11430, 11432, 11433, 11434, 11435, 11436, 11001, 11425
Queens	409 Southeast Queens	11004, 11005, 11411, 11413, 11422, 11426, 11427, 11428, 11429
Queens	410 Rockaway	11691, 11692, 11693, 11694, 11695, 11697
Staten Island	501 Port Richmond	10302, 10303, 10310
Staten Island	502 Stapleton - St. George	10301, 10304, 10305
Staten Island	503 Willowbrook	10314, 10311
Staten Island	504 South Beach - Tottenville	10306, 10307, 10308, 10309, 10312

© 2025 Reyes Nieva et al. *JAMA Network Open.*

eTable 2. Adjusted Odds Ratios for Laboratory Testing and Positive Test Results by Sexually Transmitted Infection and Patient Characteristics

Characteristics	Chlamydia				Gonorrhea				HIV			
	Testing		Positivity		Testing		Positivity		Testing		Positivity	
	OR (95% CI)	P-value	OR (95% CI)	P-value	OR (95% CI)	P-value	OR (95% CI)	P-value	OR (95% CI)	P-value	OR (95% CI)	P-value
**Sex**[a]		<0.001		<0.001		<0.001		<0.001		<0.001		<0.001
Female	Reference		Reference		Reference		Reference		Reference		Reference	
Male	0.62 (0.62-0.63)		1.09 (1.05-1.12)		0.63 (0.63-0.63)		3.28 (3.11-3.45)		1.16 (1.15-1.17)		5.18 (4.79-5.61)	
Unknown	0.66 (0.63-0.69)		0.79 (0.57-1.09)		0.65 (0.62-0.68)		2.31 (1.51-3.53)		1.05 (1.01-1.08)		5.13 (3.6-7.32)	
**Race or Ethnicity**[b]		<0.001		<0.001		<0.001		<0.001		<0.001		<0.001
Asian or Pacific Islander	0.98 (0.97-0.99)		1.00 (0.93-1.08)		0.95 (0.94-0.95)		0.50 (0.43-0.58)		1.03 (1.02-1.04)		0.33 (0.26-0.42)	
Black or African American	1.06 (1.05-1.07)		3.42 (3.24-3.61)		1.06 (1.05-1.07)		4.08 (3.73-4.46)		1.18 (1.17-1.19)		3.18 (2.82-3.57)	
Hispanic or Latino	1.13 (1.12-1.14)		2.32 (2.19-2.46)		1.13 (1.12-1.14)		1.34 (1.21-1.49)		1.23 (1.22-1.24)		1.74 (1.53-1.99)	
White	Reference		Reference		Reference		Reference		Reference		Reference	
Other race[c]	1.01 (1.00-1.02)		1.83 (1.72-1.96)		0.99 (0.98-1.00)		1.44 (1.28-1.61)		1.03 (1.02-1.04)		1.45 (1.25-1.68)	
Unknown	1.17 (1.16-1.18)		1.86 (1.76-1.98)		1.14 (1.13-1.15)		1.34 (1.21-1.48)		1.18 (1.17-1.19)		1.36 (1.19-1.54)	
**Age Group**		<0.001		<0.001		<0.001		<0.001		<0.001		<0.001
18-19 years	Reference		Reference		Reference		Reference		Reference		Reference	
20-24 years	0.55 (0.53-0.56)		0.70 (0.66-0.74)		0.50 (0.49-0.52)		0.75 (0.67-0.83)		1.04 (1.02-1.07)		2.05 (1.48-2.84)	
25-29 years	0.63 (0.62-0.64)		0.38 (0.35-0.40)		0.58 (0.57-0.59)		0.54 (0.49-0.60)		1.11 (1.09-1.14)		3.33 (2.43-4.57)	
30-34 years	0.45 (0.44-0.45)		0.20 (0.19-0.21)		0.41 (0.40-0.42)		0.36 (0.32-0.40)		1.10 (1.07-1.12)		3.94 (2.88-5.39)	
35-39 years	0.52 (0.51-0.53)		0.12 (0.11-0.14)		0.47 (0.47-0.48)		0.25 (0.22-0.29)		1.05 (1.02-1.07)		3.49 (2.54-4.78)	
40-44 years	0.36 (0.35-0.37)		0.08 (0.07-0.09)		0.33 (0.32-0.33)		0.17 (0.15-0.20)		0.86 (0.84-0.88)		2.81 (2.03-3.88)	
45-49 years	0.40 (0.39-0.41)		0.06 (0.05-0.06)		0.37 (0.36-0.37)		0.12 (0.10-0.14)		0.78 (0.76-0.79)		3.09 (2.23-4.27)	
50-54 years	0.27 (0.26-0.27)		0.04 (0.04-0.05)		0.24 (0.24-0.25)		0.07 (0.06-0.09)		0.67 (0.65-0.69)		3.19 (2.31-4.40)	
55-59 years	0.27 (0.27-0.28)		0.03 (0.02-0.03)		0.24 (0.24-0.25)		0.06 (0.05-0.08)		0.65 (0.63-0.66)		3.24 (2.35-4.48)	
60-64 years	0.19 (0.19-0.20)		0.01 (0.01-0.02)		0.17 (0.17-0.17)		0.02 (0.02-0.03)		0.53 (0.52-0.54)		2.29 (1.64-3.18)	
65+ years	0.08 (0.08-0.08)		0.005 (0.004-0.006)		0.07 (0.06-0.07)		0.01 (0.01-0.01)		0.39 (0.38-0.40)		1.07 (0.77-1.50)	
**Borough of Residence**		<0.001		<0.001		<0.001		<0.001		<0.001		<0.001
Bronx	Reference		Reference		Reference		Reference		Reference		Reference	
Brooklyn	1.02 (1.01-1.03)		0.87 (0.83-0.91)		1.04 (1.03-1.05)		0.88 (0.81-0.95)		1.04 (1.03-1.05)		0.67 (0.61-0.74)	
Manhattan	0.92 (0.91-0.93)		0.92 (0.87-0.96)		0.94 (0.93-0.95)		1.19 (1.09-1.29)		0.94 (0.93-0.95)		0.83 (0.74-0.92)	
Queens	0.93 (0.92-0.94)		0.98 (0.93-1.03)		0.97 (0.96-0.98)		0.80 (0.72-0.88)		0.95 (0.94-0.96)		0.50 (0.45-0.56)	
Staten Island	0.90 (0.89-0.92)		1.00 (0.93-1.09)		0.96 (0.95-0.98)		1.26 (1.10-1.44)		0.78 (0.77-0.79)		0.55 (0.44-0.68)	

© 2025 Reyes Nieva et al. *JAMA Network Open.*

Characteristics	Chlamydia				Gonorrhea				HIV			
	Testing		Positivity		Testing		Positivity		Testing		Positivity	
	OR (95% CI)	P-value	OR (95% CI)	P-value	OR (95% CI)	P-value	OR (95% CI)	P-value	OR (95% CI)	P-value	OR (95% CI)	P-value
**Area-Based Poverty Level**[d]		<0.001		<0.001		<0.001		<0.001		<0.001		<0.001
Low (<10% below FPT)	Reference		Reference		Reference		Reference		Reference		Reference	
Medium (10% to <20%)	0.98 (0.97-0.98)		1.12 (1.07-1.17)		0.93 (0.93-0.94)		1.31 (1.20-1.43)		0.99 (0.99-1.00)		1.56 (1.38-1.76)	
High (20% to <30%)	1.00 (0.99-1.01)		1.36 (1.29-1.43)		0.97 (0.96-0.98)		1.61 (1.45-1.77)		1.00 (0.99-1.01)		1.49 (1.30-1.71)	
Very high (30% to 100%)	0.94 (0.93-0.95)		1.41 (1.33-1.50)		0.90 (0.89-0.91)		1.91 (1.72-2.12)		1.01 (1.00-1.02)		1.79 (1.55-2.07)	
Unknown	1.07 (0.97-1.18)		1.29 (0.71-2.35)		1.04 (0.94-1.15)		1.16 (0.51-2.64)		1.02 (0.93-1.12)		3.13 (1.39-7.06)	

Abbreviations: ABPL, Area-based Poverty Level. CI, Confidence Interval. FPT, federal poverty threshold. HIV, human immunodeficiency virus. OMOP, Observational Medical Outcomes Partnership. OR, Odds Ratio. CDM, Common Data Model. [a]The OMOP CDM uses the term “gender” while source data were based on sex; source values may represent sex assigned at birth and/or administrative sex used for billing purposes (which may reflect gender identity, not sex assigned at birth). [b]All categories other than "Hispanic or Latino" are exclusively non-Hispanic or Latino (e.g., non-Hispanic White). [c]“Other Race” category is derived directly from reporting of “Other”, “Other race”, or similar in the source data. [d]ABPL categorized as low (<10% of residents in ZIP code tabulation area living below the FPT), medium (10% to <20%), high (20 to <30%), and very high (30 to 100%).

© 2025 Reyes Nieva et al. *JAMA Network Open.*

eTable 3. Unadjusted Odds Ratios for Laboratory Testing and Positive Test Results by Sexually Transmitted Infection and Patient Characteristics

Characteristics	Chlamydia				Gonorrhea				HIV			
	Testing		Positivity		Testing		Positivity		Testing		Positivity	
	OR (95% CI)	P-value	OR (95% CI)	P-value	OR (95% CI)	P-value	OR (95% CI)	P-value	OR (95% CI)	P-value	OR (95% CI)	P-value
**Sex[a]**		<0.001		<0.001		<0.001		<0.001		<0.001		<0.001
Female	Reference		Reference		Reference		Reference		Reference		Reference	
Male	0.59 (0.59-0.60)		0.94 (0.91-0.97)		0.60 (0.60-0.60)		2.85 (2.70-3.01)		1.11 (1.10-1.11)		4.96 (4.59-5.36)	
Unknown	0.33 (0.31-0.34)		0.25 (0.18-0.35)		0.31 (0.30-0.32)		0.74 (0.47-1.15)		0.69 (0.67-0.71)		3.26 (2.30-4.62)	
**Race or Ethnicity[b]**		<0.001		<0.001		<0.001		<0.001		<0.001		<0.001
Asian or Pacific Islander	1.00 (0.99-1.01)		1.01 (0.94-1.09)		0.97 (0.96-0.98)		0.52 (0.44-0.61)		1.07 (1.06-1.08)		0.34 (0.27-0.43)	
Black or African American	1.17 (1.16-1.18)		4.30 (4.07-4.54)		1.16 (1.15-1.17)		5.30 (4.86-5.79)		1.27 (1.26-1.28)		3.80 (3.39-4.25)	
Hispanic or Latino	1.33 (1.32-1.34)		3.42 (3.23-3.62)		1.33 (1.32-1.34)		1.92 (1.74-2.12)		1.36 (1.35-1.38)		2.11 (1.87-2.38)	
White	Reference		Reference		Reference		Reference		Reference		Reference	
Other race[c]	1.14 (1.13-1.15)		2.42 (2.27-2.58)		1.11 (1.10-1.13)		1.94 (1.73-2.17)		1.22 (1.20-1.23)		1.71 (1.48-1.97)	
Unknown	1.29 (1.27-1.30)		2.34 (2.21-2.48)		1.25 (1.24-1.26)		1.75 (1.58-1.94)		1.18 (1.17-1.19)		1.62 (1.42-1.84)	
**Age Group**		<0.001		<0.001		<0.001		<0.001		<0.001		<0.001
18-19 years	Reference		Reference		Reference		Reference		Reference		Reference	
20-24 years	0.52 (0.51-0.54)		0.67 (0.63-0.71)		0.47 (0.46-0.48)		0.69 (0.62-0.78)		1.03 (1.01-1.06)		1.82 (1.31-2.52)	
25-29 years	0.64 (0.63-0.66)		0.35 (0.33-0.37)		0.59 (0.57-0.60)		0.49 (0.43-0.55)		1.10 (1.07-1.12)		2.94 (2.14-4.03)	
30-34 years	0.42 (0.41-0.43)		0.18 (0.17-0.19)		0.38 (0.37-0.39)		0.32 (0.28-0.36)		1.08 (1.06-1.11)		3.53 (2.58-4.82)	
35-39 years	0.52 (0.51-0.53)		0.11 (0.10-0.12)		0.47 (0.46-0.48)		0.23 (0.19-0.26)		1.03 (1.01-1.06)		3.15 (2.30-4.33)	
40-44 years	0.34 (0.33-0.35)		0.08 (0.07-0.08)		0.30 (0.29-0.31)		0.16 (0.14-0.19)		0.86 (0.84-0.88)		2.63 (1.90-3.63)	
45-49 years	0.39 (0.38-0.40)		0.05 (0.04-0.06)		0.35 (0.34-0.36)		0.11 (0.09-0.13)		0.77 (0.75-0.79)		2.97 (2.15-4.10)	
50-54 years	0.25 (0.25-0.26)		0.04 (0.03-0.05)		0.22 (0.22-0.23)		0.07 (0.06-0.09)		0.67 (0.65-0.68)		3.26 (2.37-4.50)	
55-59 years	0.26 (0.25-0.26)		0.03 (0.02-0.03)		0.22 (0.22-0.23)		0.07 (0.05-0.09)		0.64 (0.63-0.66)		3.43 (2.49-4.73)	
60-64 years	0.18 (0.18-0.18)		0.01 (0.01-0.02)		0.16 (0.15-0.16)		0.02 (0.02-0.04)		0.52 (0.51-0.54)		2.33 (1.67-3.24)	
65+ years	0.07 (0.07-0.08)		0.004 (0.003-0.005)		0.06 (0.06-0.06)		0.01 (0.01-0.01)		0.38 (0.37-0.39)		0.98 (0.70-1.36)	
**Borough of Residence**		<0.001		<0.001		<0.001		<0.001		<0.001		<0.001
Bronx	Reference		Reference		Reference		Reference		Reference		Reference	
Brooklyn	0.93 (0.92-0.94)		0.63 (0.60-0.65)		0.94 (0.94-0.95)		0.67 (0.62-0.72)		0.95 (0.95-0.96)		0.54 (0.49-0.59)	
Manhattan	0.85 (0.84-0.86)		0.58 (0.56-0.61)		0.87 (0.87-0.88)		0.72 (0.66-0.78)		0.86 (0.85-0.86)		0.61 (0.55-0.67)	
Queens	0.82 (0.81-0.83)		0.54 (0.52-0.57)		0.85 (0.84-0.86)		0.39 (0.36-0.42)		0.85 (0.85-0.86)		0.32 (0.29-0.36)	
Staten Island	0.80 (0.78-0.81)		0.52 (0.48-0.56)		0.85 (0.84-0.86)		0.60 (0.52-0.68)		0.68 (0.67-0.69)		0.31 (0.26-0.38)	

Characteristics	Chlamydia				Gonorrhea				HIV			
	Testing		Positivity		Testing		Positivity		Testing		Positivity	
	OR (95% CI)	P-value	OR (95% CI)	P-value	OR (95% CI)	P-value	OR (95% CI)	P-value	OR (95% CI)	P-value	OR (95% CI)	P-value
**Area-based Poverty Level**[d]		<0.001		<0.001		<0.001		<0.001		<0.001		<0.001
Low (<10% below FPT)	Reference		Reference		Reference		Reference		Reference		Reference	
Medium (10% to <20%)	1.06 (1.05-1.07)		1.32 (1.26-1.38)		1.01 (1.00-1.02)		1.51 (1.38-1.65)		1.09 (1.08-1.09)		1.91 (1.69-2.15)	
High (20% to <30%)	1.14 (1.13-1.15)		1.77 (1.69-1.86)		1.09 (1.09-1.10)		2.28 (2.08-2.51)		1.15 (1.14-1.16)		2.30 (2.02-2.61)	
Very high (30% to 100%)	1.16 (1.15-1.17)		2.30 (2.19-2.42)		1.10 (1.09-1.11)		3.30 (3.01-3.62)		1.23 (1.22-1.24)		3.71 (3.28-4.21)	
Unknown	1.12 (1.02-1.23)		1.44 (0.79-2.63)		1.08 (0.98-1.19)		1.32 (0.56-3.14)		1.10 (1.00-1.20)		4.93 (2.20-11.10)	

Abbreviations: ABPL, Area-based Poverty Level. CI, Confidence Interval. FPT, federal poverty threshold. HIV, human immunodeficiency virus. OMOP, Observational Medical Outcomes Partnership. OR, Odds Ratio. CDM, Common Data Model. [a]The OMOP CDM uses the term "gender" while source data were based on sex; source values may represent sex assigned at birth and/or administrative sex used for billing purposes (which may reflect gender identity, not sex assigned at birth). [b]All categories other than "Hispanic or Latino" are exclusively non-Hispanic or Latino (e.g., non-Hispanic White). [c]"Other Race" category is derived directly from reporting of "Other", "Other race", or similar in the source data. [d]ABPL categorized as low (<10% of residents in ZIP code tabulation area living below the FPT), medium (10% to <20%), high (20 to <30%), and very high (30 to 100%).

© 2025 Reyes Nieva et al. *JAMA Network Open.*

eTable 4. Neighborhood-Level Spatial Autocorrelation of Laboratory Testing and Positive Test Results

Sexually transmitted infection	Testing			Positive Cases			Absolute Difference between Testing and Case Prevalence		
	Moran's I	*z-score*	*P value*	*Moran's I*	*z-score*	*P value*	*Moran's I*	*z-score*	*P value*
Chlamydia	0.213	2.157	0.02	0.205	2.063	0.02	0.453	4.332	<0.001
Gonorrhea	0.207	2.108	0.02	0.177	1.841	0.03	0.307	3.010	0.001
HIV	0.237	2.389	0.01	0.228	2.304	0.01	0.358	3.472	<0.001

© 2025 Reyes Nieva et al. *JAMA Network Open.*

eTable 5. Citywide Percentages of Laboratory Tests and Positive Results by Neighborhood

Borough	UHF Code and Neighborhood Name	Chlamydia			Gonorrhea			HIV		
		Cases	Tests	Absolute Difference	Cases	Tests	Absolute Difference	Cases	Tests	Absolute Difference
						%				
Bronx	101 Kingsbridge - Riverdale	0.7	0.7	0	0.6	0.7	-0.1	0.6	0.8	-0.2
Bronx	102 Northeast Bronx	2.0	1.4	+0.6	2.1	1.4	+0.7	2.1	1.4	+0.7
Bronx	103 Fordham - Bronx Park	3.8	3.1	+0.7	2.8	3.0	-0.2	5.8	2.8	+3.0
Bronx	104 Pelham - Throgs Neck	3.3	3.2	+0.1	2.5	3.1	-0.6	2.9	3.0	-0.1
Bronx	105 Crotona - Tremont	4.8	3.8	+1.0	5.1	3.6	+1.5	6.1	3.2	+2.9
Bronx	106 High Bridge - Morrisania	4.3	3.1	+1.2	4.7	3.0	+1.7	5.0	2.9	+2.1
Bronx	107 Hunts Point - Mott Haven	3.8	2.6	+1.2	3.8	2.5	+1.3	4.5	2.1	+2.4
Brooklyn	201 Greenpoint	1.0	1.6	-0.6	1.5	1.6	-0.1	0.7	1.6	-0.9
Brooklyn	202 Downtown - Heights - Park Slope	1.6	2.4	-0.8	2.4	2.5	-0.1	2.1	2.7	-0.6
Brooklyn	203 Bedford Stuyvesant - Crown Heights	5.9	4.5	+1.4	7.6	4.6	+3.0	7.8	4.7	+3.1
Brooklyn	204 East New York	2.1	1.4	+0.7	2.0	1.4	+0.6	1.8	1.3	+0.5
Brooklyn	205 Sunset Park	4.2	4.2	0.0	3.1	4.2	-1.1	3.2	4.3	-1.1
Brooklyn	206 Borough Park	1.9	3.3	-1.4	1.2	3.3	-2.1	1.7	3.8	-2.1
Brooklyn	207 East Flatbush - Flatbush	4.3	3.6	+0.7	5.1	3.6	+1.5	6.3	3.6	+2.7
Brooklyn	208 Canarsie - Flatlands	2.3	2.0	+0.3	1.8	2.0	-0.2	2.0	2.3	-0.3
Brooklyn	209 Bensonhurst - Bay Ridge	1.2	2.3	-1.1	0.7	2.3	-1.6	1.2	2.6	-1.4
Brooklyn	210 Coney Island - Sheepshead Bay	1.9	3.1	-1.2	1.6	2.9	-1.3	1.7	3.0	-1.3
Brooklyn	211 Williamsburg - Bushwick	4.0	3.5	+0.5	4.9	3.5	+1.4	2.8	3.0	-0.2
Manhattan	301 Washington Heights - Inwood	4.2	3.5	+0.7	4.0	3.4	+0.6	3.8	3.3	+0.5
Manhattan	302 Central Harlem - Morningside Heights/ Manhattan	3.1	2.5	+0.6	5.5	2.5	+3.0	3.8	2.3	+1.5
Manhattan	303 East Harlem	2.6	1.8	+0.8	3.0	1.7	+1.3	2.5	1.9	+0.6
Manhattan	304 Upper West Side	1.0	1.5	-0.5	1.2	1.5	-0.3	1.7	1.7	0.0
Manhattan	305 Upper East Side	1.0	1.8	-0.8	1.1	1.9	-0.8	0.9	1.9	-1.0
Manhattan	306 Chelsea - Clinton	1.8	2.4	-0.6	4.1	2.4	+1.7	4.4	2.2	+2.2
Manhattan	307 Gramercy Park - Murray Hill	1.0	1.5	-0.5	1.1	1.5	-0.4	1.2	1.4	-0.2

© 2025 Reyes Nieva et al. *JAMA Network Open.*

Borough	UHF Code and Neighborhood Name	Chlamydia			Gonorrhea			HIV		
		Cases	Tests	Absolute Difference	Cases	Tests	Absolute Difference	Cases	Tests	Absolute Difference
						%				
Manhattan	308 Greenwich Village - SoHo	0.4	0.8	-0.4	0.5	0.8	-0.3	0.3	0.7	-0.4
Manhattan	309 Union Square - Lower East Side	1.7	1.9	-0.2	1.8	1.9	-0.1	1.2	2.0	-0.8
Manhattan	310 Lower Manhattan	0.6	0.7	-0.1	0.7	0.7	0.0	0.5	0.7	-0.2
Queens	401 Long Island City - Astoria	2.3	2.9	-0.6	2.3	2.9	-0.6	2.0	3.0	-1.0
Queens	402 West Queens	5.1	5.9	-0.8	3.5	5.8	-2.3	3.9	6.0	-2.1
Queens	403 Flushing - Clearview	2.4	3.4	-1.0	1.4	3.4	-2.0	0.9	3.3	-2.4
Queens	404 Bayside - Little Neck	0.5	0.8	-0.3	0.3	0.8	-0.5	0.3	0.9	-0.6
Queens	405 Ridgewood - Forest Hills	1.8	2.7	-0.9	1.0	2.8	-1.8	1.2	2.4	-1.2
Queens	406 Fresh Meadows	0.9	1.1	-0.2	0.6	1.1	-0.5	0.2	1.2	-1.0
Queens	407 Southwest Queens	3.0	3.2	-0.2	1.8	3.2	-1.4	2.2	3.5	-1.3
Queens	408 Jamaica	5.2	4.1	+1.1	4.0	4.2	-0.2	4.4	4.5	-0.1
Queens	409 Southeast Queens	2.8	2.3	+0.5	2.3	2.4	-0.1	1.6	2.6	-1.0
Queens	410 Rockaway	1.6	1.2	+0.4	1.6	1.2	+0.4	1.4	1.3	+0.1
Staten Island	501 Port Richmond	1.0	0.8	+0.2	1.0	0.8	+0.2	0.6	0.7	-0.1
Staten Island	502 Stapleton - St. George	1.7	1.4	+0.3	2.3	1.4	+0.9	1.6	1.5	+0.1
Staten Island	503 Willowbrook	0.6	0.7	-0.1	0.5	0.7	-0.2	0.4	0.7	-0.3
Staten Island	504 South Beach - Tottenville	1.0	1.6	-0.6	0.9	1.7	-0.8	0.5	1.3	-0.8

Abbreviations: UHF, United Hospital Fund. Case percentages determined by the number of positive cases identified in a given neighborhood divided by the total number of positive cases found in all of New York City. Test percentages determined by the number of laboratory tests performed in a given neighborhood divided by the total number of tests performed in all of New York City. Columns labeled “Cases” and “Tests” may not total to 100% due to rounding. Absolute difference calculated by subtracting the percent of tests conducted in a given neighborhood from the percent of positive cases identified in that same neighborhood.

© 2025 Reyes Nieva et al. *JAMA Network Open.*
