# Supplementary material for: Enhanced Surveillance of Sexually Transmitted Infections to Foster a Learning Public Health System
Source: JAMA Netw Open. 2025 Jun 17;8(6):e2514308. doi: 10.1001/jamanetworkopen.2025.14308 (PMC12175029; doi:10.1001/jamanetworkopen.2025.14308)
Supplement: Supplement 2. — Data Sharing Statement [file jamanetwopen-e2514308-s002.pdf]

Data Sharing Statement

Reyes Nieva. Surveillance of HIV and Other Sexually Transmitted Infections in a Learning Public Health System. *JAMA Netw Open*. Published June 09, 2025. doi:10.1001/jamanetworkopen.2025.14308

Data
Data available: No

Additional Information
Explanation for why data not available: The electronic health record and administrative data underlying this article are not available due to restrictions to preserve patient confidentiality.
